# A Dictionary of Practical Medicine, Comprising General Pathology, the Nature and Treatment of Diseases, Morbid Structures, &c.

**Published:** 1846-05

**Authors:** 


Art. VIII.—*A Dictionary of Practical Medicine, comprising General Pathology, the Nature and Treatment of Diseases, Morbid Structures, &c.* By James Copland, M.D., F.R.S. Edited, with additions. By Charles A. Lee, M.D. New York: Harper and Brothers, No. 82, Cliff street. 1845. 8vo. Parts I to XIII.

No medical production of the age has called forth from the medical press stronger expressions of admiration than the work of which the name is given above. It is regarded with a feeling of astonishment as the production of one man. The labor required for the preparation of a work of such extent must be immense; but it is not so much the amplitude of the task as the excellence of its execution, that has extorted such general and warm applause. Where the field to be gone over was so broad it was hardly to be hoped that one mind could do justice to all the topics presented; and yet each article in the *Dictionary* is a monograph so perfect in itself that it would not be easy to improve upon it. Thirteen numbers of the Dictionary have now been issued, and seven more are expected to complete the work, which will be embodied in three large octavo volumes, with small print and double columns, and is offered to subscribers at a very reasonable price.

The value of a work like the above to the practitioner who must often be going back to the authorities in his profession, it would be difficult to exaggerate. Here, in a brief compass, compressed into three volumes, and alphabetically arranged, he has a compendium of medical science and practice, by a physician of profound learning and matured experience. The opinions it expresses are such as he may generally adopt; the practice such as the experience of a long life, laboriously spent among disease, sanctions; it exhibits medical science as it was the day the articles came from the author's pen; and by the ample references at the end of each article puts it in the power of the reader to prosecute his researches to any desired extent.

Dr. Lee, the American editor, is known to his countrymen as the laborious editor of several works, and we believe the truth of our remark will be concurred in by all, that he has touched no work which has not come from his hands enriched and improved. The office which Dr. Lee has assigned himself in his present undertaking, is to supply whatever may have been added to our progressive science since Dr. Copland sent his articles to the press, and especially to add what has been done in practical medicine by American physicians. Thus far he appears to us to have performed that office with impartiality and judgment. He has ransacked our medical literature, and nothing seems to have escaped him. Appended to Dr. Copland's "bibliography," will be found references by Dr. Lee to every medical journal extant in the Union, and nearly every leading medical paper that has been written by his countrymen.

We need not insist upon the desirableness of such a Dictionary as this. Every practitioner who can spare the means will possess himself of it. Mr. James Maxwell, or Mr. F. W. Prescott, of this city, will receive and transmit orders for it, or for any other medical work published in this country. They keep always on hand an excellent stock of standard medical books.

